# Meiotic outcome in two carriers of Y autosome reciprocal translocations: selective elimination of certain segregants

**DOI:** 10.1186/s13039-017-0303-y

**Published:** 2017-02-02

**Authors:** Harita Ghevaria, Roy Naja, Sioban SenGupta, Paul Serhal, Joy Delhanty

**Affiliations:** 1 Preimplantation Genetics Group, Institute for Women’s Health, University College London, 86-96 Chenies Mews, London, WC1E 6HX UK; 2 The Centre for Reproductive and Genetic Health, 230-232 Great Portland Street, London, W1W 5QS UK

**Keywords:** Y-autosome Translocation, Meiosis, Segregation, Infertility

## Abstract

**Background:**

Reciprocal Y autosome translocations are rare but frequently associated with male infertility. We report on the meiotic outcome in embryos fathered by two males with the karyotypes 46,X,t(Y;4)(q12;p15.32) and 46,X,t(Y;16)(q12;q13). The two couples underwent preimplantation genetic diagnosis (PGD) enabling determination of the segregation types that were compatible with fertilization and preimplantation embryo development. Both PGD and follow up analysis were carried out via fluorescence in situ hybridization (FISH) or array comparative genomic hybridization (aCGH) allowing the meiotic segregation types to be determined in a total of 27 embryos.

**Results:**

Interestingly, it was seen that the number of female embryos resulting from alternate segregation with the chromosome combination of X and the autosome from the carrier gamete differed from the corresponding balanced males with derivative Y and the derivative autosome by a ratio of 7:1 in each case (*P* = 0.003) while from the adjacent-1 mode of segregation, the unbalanced male embryos with the combination of der Y and the autosome were seen in all embryos from couple A and in couple B with the exception of one embryo only that had the other chromosome combination of X and derivative autosome (*P* = 0.011). In both cases the deficit groups have in common the der autosome chromosome that includes the segment Yq12 to qter.

**Conclusion:**

The most likely explanation may be that this chromosome is associated with the X chromosome at PAR2 (pseudoautosomal region 2) in the sex-body leading to inactivation of genes on the autosomal segment that are required for the meiotic process and that this has led to degeneration of this class of spermatocytes during meiosis.

## Background

Reciprocal translocations between the Y chromosome and an autosome are rare and highly associated with male infertility. From a review of 22 cases of balanced Y-autosome translocations, it became clear that if the break occurs within the Yq critical segment (AZF) or near the primary pseudoautosomal segment (PAR1) and hence the SRY gene, germ cell maturation may be severely damaged, resulting in azoospermia or severe oligozoospermia in the carrier males [1, 2].

However, translocations involving breaks in the Yq12 heterochromatic region also frequently lead to infertility. During normal male meiosis, recombination normally takes place between the X and Y chromosomes at PAR 1 located at the tips of both the Xp and Yp and the remainder of the chromosome remains unsynapsed. However, synapsis may also occur at PAR 2, located at the terminal region of the long arms of X & Y. Thus in the first meiotic metaphase the two sex chromosomes form the XY-body or the sex-body, which is genetically inactivated during the pachytene stage of meiosis. The formation of the sex-body enables normal meiotic progression even in the presence of unsynapsed regions [3]. In the case of a Y-autosome translocation, if a translocated chromosome is associated with the sex-body, inactivation may extend to the autosomal segment. If this segment houses pachytene critical genes the consequence may be degeneration of most of the spermatocytes after the pachytene stage [4].

A few clinical investigations involving Y-autosome translocations with familial histories have been reported previously in the literature. An infertile man with severe oligoasthenospermia was found to have the karyotype 46,X,t(1;Y) (q11;q11). His father who was proved to have the same translocation, also had two daughters and one other son [5]. The report of the family investigated by (Sklower Brooks et al. in 1998), describes a couple where the male had the abnormal karyotype 46,X,t(Y;8)(q12;p21.3) and the woman had reported a third miscarriage involving the t(Y;8) translocation. This couple also had a normal daughter. The man had four brothers and two sisters. The investigation revealed that their deceased father must have carried the abnormal karyotype which was passed on to four of his sons in a balanced state and in an unbalanced state to the remaining one [6]

We present here the meiotic outcome for two carriers of reciprocal Y-autosome translocations ascertained after a total of six cycles of preimplantation genetic diagnosis (PGD). The data provide evidence for the selective elimination of certain gametic segregants.

## Methods

### Patient Details

Two couples, where the male patient was a carrier of a reciprocal Y-autosome translocation, underwent six cycles of PGD between the years 2014–2015. The necessary IVF (in vitro fertilization) treatment took place at the Centre for Reproductive and Genetic Health (CRGH). All genetic diagnoses were carried out at UCL Centre for PGD with the exception of those for one cycle of treatment for couple B. The karyotype of the carrier patients along with their reproductive histories is shown in Table 1.

**Table 1:** Reproductive histories and karyotypes of the two carriers of reciprocal Y-autosome translocations

	Type of ART used	Male Karyotype	Sperm Parameters	Reproductive History
Couple A	ICSI	46,X,t(Y;4)(q12;p15.32)	Severe oligozoospermia	Primary infertility No previous pregnancies
Couple B	ICSI	46,X,t(Y;16)(q12;q13)	Severe oligozoospermia	Primary infertility No previous pregnancies

*ART* Assisted Reproductive Technology, *ICSI* Intracytoplasmic Sperm Injection

### IVF and PGD

For both couples, fresh semen was retrieved on the day of the egg collection. Routine semen analysis revealed sub-optimal semen parameters and both male carriers presented with severe oligozoospermia. Hence during the IVF treatment, intracytoplasmic sperm injection (ICSI) was the chosen method of insemination (Table 1).

Three PGD cycles were carried out for each of the two couples. For couple A, all three cycles were performed using Fluorescence In Situ Hybridisation (FISH) at cleavage stage (day 3 of embryo development) where one or two blastomeres were biopsied for diagnosis. For couple B, one cycle was performed using FISH at cleavage stage and the remaining two using array comparative genomic hybridisation (aCGH) at the blastocyst stage (day 5 or 6 of embryo development) where diagnosis was performed on a few trophectoderm (TE) cells.

### Analysis by FISH

For cycles where PGD was performed by FISH, a patient specific protocol was developed and optimized on lymphocytes from peripheral blood prior to the clinical application of PGD on single blastomeres. Ideograms were constructed in order to choose the appropriate probes based on the position of the breakpoint for each translocation. The details of probes used for both the diagnosis and follow up analyses are given in Table 2. In these two cases, the probe strategies selected could distinguish normal female embryos from males chromosomally balanced for the translocation.

**Table 2:** Probes used in FISH analysis for couples A and B.

	Male karyotype	Probes used for FISH
Couple A	46,X,t(Y;4)(q12;p15.32)	1st Round: CEP 4 (SA); Tel 4p (SG); CEP X (SO) 2nd Round: ^a^CEP Y (DYZ1) (SA); Tel Xq/Yq (SO)
Couple B	46,X,t(Y;16)(q12;q13)	1st Round: Tel Xq/Yq (SO), CEP 16 (SA); ^b^Tel 16q (SG) 2nd Round: ^c^CEP Y (DYZ3) (SO), CEP X (SG).

*SA* Spectrum Aqua, *SG* Spectrum Green, *SO* Spectrum Orange

All probes were from Abbott Molecular, UK unless stated

^a^CEP Y (DYZ1) : Cytogenetic Location Yq12, Satellite III DNA

^b^ = Kreatech FISH Probes, Leica Biosystems, UK

^c^CEP Y (DYZ3) : Cytogenetic Location Yp11.1-q11.1, Alpha Satellite DNA

Fluorescence In Situ Hybridisation was performed in two rounds of hybridisation (Table 2). The FISH protocol was carried out as described previously with slight modifications [7]. Microscopic analysis and scoring of FISH signals were carried out using an epifluorescence Olympus microscope (Olympus BX 40, London, UK). FISH signals were scored according to [8]

### Analysis by array-CGH

For both diagnosis and follow up cells were subjected to aCGH using 24Sure + arrays (BlueGnome Ltd., Fulbourn, Cambridge UK, now Illumina). Prior to the aCGH, whole genome amplification was carried out using the Sureplex^TM^ amplification kit (BlueGnome Ltd., Fulbourn, Cambridge UK, now Illumina). Amplification efficiency was assessed by gel electrophoresis. Array-CGH was carried out according to the manufacturer’s protocol with slight modifications. Images were scanned and analysed using BlueFuse Multi software (BlueGnome Ltd, now Illumina). Details for both the protocol and analysis has been described elsewhere [9].

### Follow up analysis in embryos obtained on day 5–7 post-fertilisation

After transfer of embryos diagnosed as unaffected, the untransferred embryos were available for confirmation of diagnosis and follow up. Where diagnosis was by FISH, embryos were either subjected to follow up using the same strategy or via aCGH. If PGD was carried out using aCGH, then follow up was also via aCGH. The diagnostic and follow up result using aCGH also revealed aneuploidies of other unrelated chromosomes; these are not reported here.

### Meiotic segregation analysis

The segregation mode at meiosis was recorded for each embryo after follow up analysis. If no follow up information was available, or if the embryo had been transferred, then the segregation mode was deduced from the PGD results obtained on the biopsied cells (day 3 or 5). This was done in order to determine the contributions of the chromosomes involved in the translocation by the different male gametes.

### Statistical Analysis

The relative frequencies of combinations of chromosomal constitutions for the alternate and the adjacent-1 segregation products deduced from the embryos were analysed using the Chi-Square for Goodness of Fit test. *P* < 0.05 was considered significant. *P* < 0.01 was considered highly significant.

## Results

### Results from the follow up analysis of untransferred embryos

**Fig. 1:**
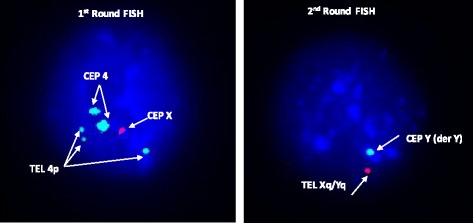
FISH image of an embryonic blastomere from the untransferred embryo no. 10 (Table 3) belonging to couple A with karyotype 46,X,t(Y;4)(q12;p15.32). The FISH signal pattern is of an unbalanced male embryo consistent with 2 × CEP 4 (SA), 3 × Tel 4p(SG), 1 × CEP X(SO) in the 1st round and 1 × CEP Y (der Y) (SA), 1 × Tel Xq/Yq (SO) in the second round of hybridisation. The expected FISH signals pattern for an embryo to be a balanced male would be 2 × CEP 4 (SA), 2 × Tel 4p(SG), 1 × CEP X(SO) in the 1^st^ round of hybridisation and 1 × CEP Y(der Y)(SA), 2 × Tel Xq/Yq (SO) in the 2^nd^ round. Meiotic segregation analysis revealed that chromosomes der Y and 4 was the contribution from the male gamete

For couple A, follow up analysis on day 5–7 was carried out on a total of 10 untransferred embryos of which 7 gave conclusive results (Table 3). Four embryos (C1E6, C1E9, C1E10, C1E12) diagnosed as unbalanced for the translocation on day 3 were confirmed as so after follow up by FISH. Follow up of the remaining three embryos by aCGH gave results showing two embryos (C1E2, C1E3) (that had no result with PGD), to be female but with additional aneuploidies unrelated to the chromosomes involved in the translocation and confirmed one (C3E3) as an unbalanced male embryo with additional aneuploidy (Table 3). In addition, results from PGD were used for nine embryos, making a total of 16 for which segregation analysis could be attempted. Figure 1 shows the FISH result of the follow up analysis of an untransferred embryo from couple A.

**Table 3:** Summary of the follow up results of embryos from three PGD cycles performed for couple A with a male karyotype 46,X,t(Y;4)(q12;p15.32)

PGD cycle no. / Embryo no.	Follow up method	Day 5–7 Follow up result (Diagnostic result where follow up result not available)	Meiotic segregation (stage determined)	Chromosomes contributed by carrier parent	Embryo grade on follow up
C1 E2	aCGH	Female embryo with additional aneuploidies	Alternate (follow up)	X and 4	cavitating morula
C1 E3	aCGH	Female embryo with multiple chromosome abnormalities	Alternate (follow up)	X and 4	pre-morula
C1 E4	n/a	Embryo transferred (normal female embryo)	Alternate (diagnosis)	X and 4	7 cells 2+
C1 E6	FISH	Male unbalanced for translocation	Adjacent-1 (follow up)	der Y and 4	blastocyst
C1 E9	FISH	Male unbalanced for translocation	Adjacent-1 (follow up)	der Y and 4	degenerating embryo
C1 E10	FISH	Male unbalanced for translocation	Adjacent-1 (follow up)	der Y and 4	hatched blastocyst
C1 E12	FISH	Male unbalanced for translocation	Adjacent-1 (follow up)	der Y and 4	morula
C2 E1	n/a	Embryo transferred (normal female embryo)	Alternate (diagnosis)	X and 4	pre-morula
C2 E2	FISH	No result (Female unbalanced for translocation)	Unknown segregation (diagnosis)	-	blastocyst
C2 E3	FISH	No result (Male unbalanced for translocation)	3:1 (diagnosis)	der Y	blastocyst
C2 E4	FISH	No result (Male; mosaic)	Unknown segregation (diagnosis)	-	blastocyst
C3 E1	n/a	Embryo cryopreserved (^a^aCGH – Male embryo with no gains or losses detected)	Alternate (diagnosis)	der Y and der 4	blastocyst
C3 E2	n/a	Embryo cryopreserved (^a^aCGH result – normal female embryo with no gains or losses detected)	Alternate (diagnosis)	X and 4	blastocyst
C3 E3	aCGH	Male embryo unbalanced for the translocation, with additional aneuploidies	Adjacent-1 (follow up)	der Y and 4	morula
C3 E4	n/a	Embryo transferred (normal female embryo)	Alternate (diagnosis)	X and 4	pre-morula
C3 E5	n/a	Embryo cryopreserved- (^a^aCGH result – normal female embryo with no gains or losses detected)	Alternate (diagnosis)	X and 4	blastocyst

*C* PGD cycle no, *E* Embryo no

^a^Diagnostic aCGH result using 24Sure + arrays after a re-biopsy at blastocyst stage on day 6 of embryo development

For couple B, follow up analysis was carried out on a total of 11 embryos of which 10 gave conclusive results (Table 4). Of the embryos followed up by FISH from cycle 1, four (C1E4, C1E5, C1E6, C1E8) were confirmed as unbalanced for the translocation. The remaining two embryos were characterised as a normal female (C1E1) and a balanced male (C1E3). In addition, follow up of four embryos from cycle 3 (initially diagnosed and followed up by aCGH) confirmed three embryos as females (C3E1, C3E3, C3E5) and one as male, unbalanced for the translocation (C3E4). For four embryos use was made of the diagnostic results giving segregation analysis results for a total of 14 embryos. One embryo gave no result (C1E2) either diagnostically or on follow up (Table 4).

**Table 4:** Summary of the follow up results of embryos from three PGD cycles performed for couple B with a male karyotype 46,X,t(Y;16)(q12;q13)

PGD cycle no./ Embryo no.	Follow up method	Day 6 Follow up result (Diagnostic result where follow up result not available)	Meiotic segregation (stage determined)	Chromosomes contributed by carrier parent	Embryo grade on follow up
C1 E1	FISH	Normal female	Alternate (follow up)	X and 16	cavitating morula
C1 E2	FISH	No result	No result	No result	pre-morula
C1 E3	FISH	Male balanced for the translocation	Alternate (follow up)	der Y and der 16	blastocyst
C1 E4	FISH	Abnormal	Unknown (follow up)	-	blastocyst
C1 E5	FISH	Male unbalanced for the translocation	Adjacent-1 (follow up)	der Y and 16	blastocyst
C1 E6	FISH	Female unbalanced for the translocation	Adjacent-1 (follow up)	X and der 16	blastocyst
C1 E7	n/a	Embryo transferred (normal female embryo)	Alternate (diagnosis)	X and 16	blastocyst
C1 E8	FISH	Male unbalanced for the translocation	Adjacent-1 (follow up)	der Y and 16	blastocyst
C2 E1	n/a	Embryo transferred (normal female embryo)	Alternate (diagnosis)	X and 16	blastocyst
C2 E2	n/a	No result (Male unbalanced for the translocation)	Adjacent-1 (diagnosis)	der Y and 16	blastocyst
C3 E1	aCGH	Normal Female	Alternate (follow up)	X and 16	blastocyst
C3 E2	n/a	Embryo transferred (normal female embryo)	Alternate (diagnosis)	X and 16	blastocyst
C3 E3	aCGH	Female embryo with additional aneuploidy	Alternate (follow up)	X and 16	blastocyst
C3 E4	aCGH	Male unbalanced for the translocation with additional aneuploidy	Adjacent-1 (assumed) (follow up)	der Y and 16	blastocyst
C3 E5	aCGH	Female embryo with additional aneuploidy	Alternate (follow up)	X and 16	blastocyst

n/a – embryo not available for follow up. The diagnostic results of cycle 3 were available from Reprogenetics,UK

### Meiotic segregation analysis

Analysis of meiotic segregation was performed for 30 embryos in total. Overall, the analysis performed on all the embryos for rearrangements involving t(Y;4) and t(Y;16) revealed alternate segregation (53%) as the most frequent mode followed by adjacent-1 (33%) and 3:1 (3%). In three embryos (10%) a segregation pattern could not be determined. For both rearrangements no embryos resulting from adjacent-2 segregation were found.

**Fig. 2:**
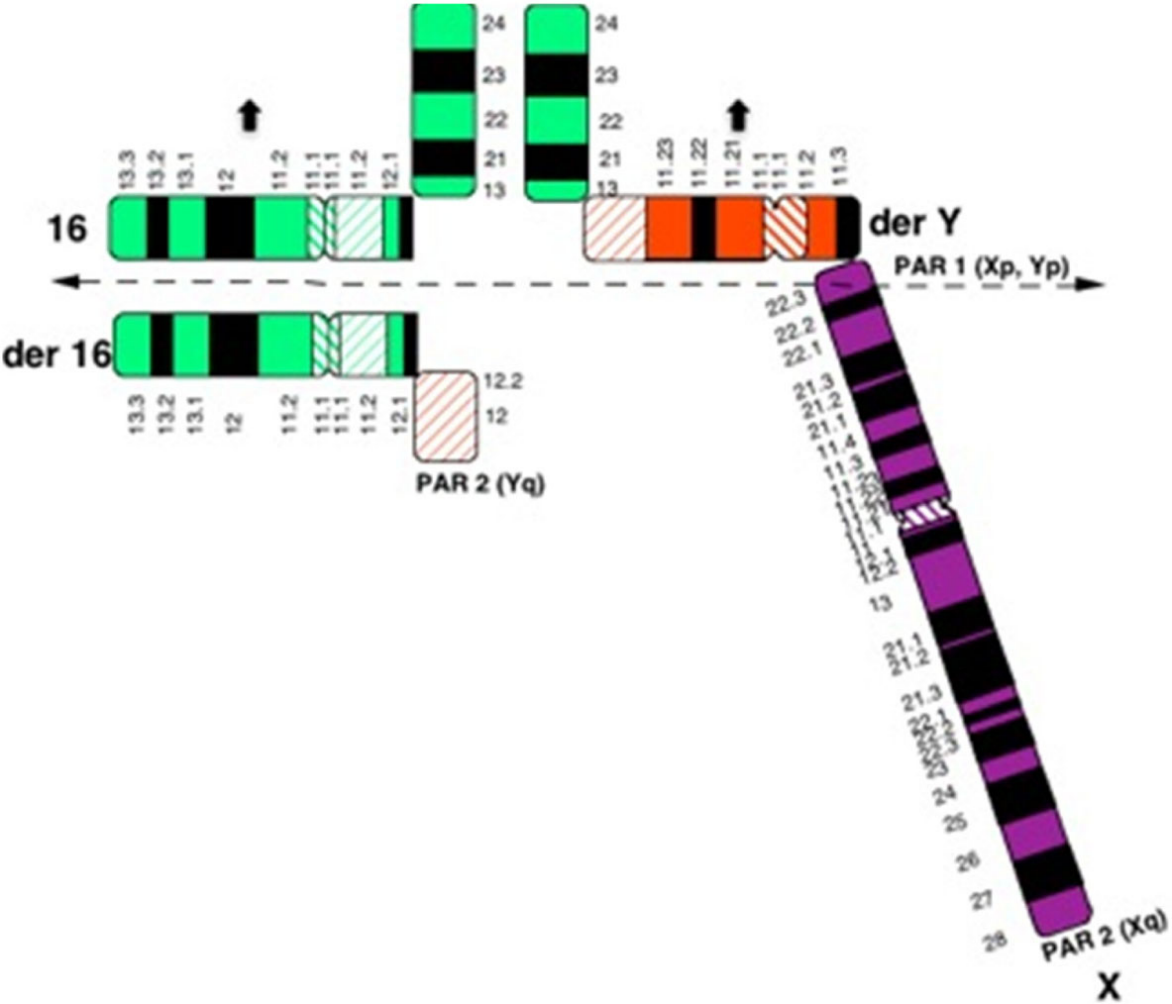
Presumed configuration of the pachytene quadrivalent at meiosis I in the gametes of the male carrier of 46,X,t(Y;16)(q12;q13), couple B. The dotted line is the adjacent-1 segregation line. Black arrows indicate the two chromosomes (der Y and 16) that were passed on to the majority of the unbalanced embryos observed after PGD in couple B

Meiotic segregation outcomes obtained for the 16 embryos belonging to couple A showed eight embryos resulting from alternate segregation; five from adjacent-1 segregation and one from 3:1 segregation (analysis based on day 3 biopsy result). A segregation pattern could not be determined for two embryos. Similarly for couple B, meiotic segregation analysis for 14 embryos was carried out. Eight embryos resulted from alternate segregation and five from adjacent-1 segregation. In this case, the segregation pattern could not be determined for one embryo. An example of the presumed pachytene quadrivalent of a Y-autosome translocation carrier is shown (Fig. 2).

Detailed analysis of the chromosome contributions from the carrier gamete in both the reciprocal Y-autosome translocation carriers showed interesting outcomes. Firstly, it was seen that the number of female embryos resulting from alternate segregation with the chromosome combination of X and the autosome (4 or 16) from the carrier gamete, was found to be far higher than the corresponding number of embryos characterised as balanced males with derivative Y and the derivative autosome, a ratio of 7:1 in each case. This observed outcome of alternate segregation deviated highly significantly (*P* = 0.003) from the expected 1:1 ratio (Table 5). Secondly, from the adjacent-1 mode of meiotic segregation, the unbalanced male embryos with the combination of der Y and the autosome were seen in all embryos from couple A and in couple B with the exception of one embryo only that had the other chromosome combination of X and derivative autosome. Again this observed outcome of adjacent-1 segregation deviated significantly (*P* = 0.011) from the expected 1:1 ratio (Table 5).

**Table 5:** Details of meiotic segregation patterns of embryos from the two reciprocal Y-autosome translocation carriers following alternate or adjacent- 1 separation

Mode of segregation	Embryo characterization with respect to the translocation	Combination of chromosomes	Embryos possessing the combination of the segregation mode for the chromosomal abnormality		Observed no. of embryos	Expected no. of embryos	Statistical Significance
			t (Y;4)	t (Y;16)			
Alternate segregation	Normal Female	X and 4/16	7 embryos	7 embryos	14	8	Deviation from 1:1 ratio, *P* = 0.003
	Balanced Male	der Y and der 4/16	1 embryo	1 embryo	2	8	
Adjacent −1	Unbalanced Male	der Y and 4/16	5 embryos	4 embryos	9	5	Deviation from 1:1 ratio, *P* = 0.011
	Unbalanced Male	X and der 4/16	0	1 embryo	1	5	

## Discussion

We have reported the detailed analysis of the translocated chromosome constitution of the embryos generated by PGD in two couples where the male partner has a Y- autosome translocation. This information has provided a rare opportunity to assess the translocation segregation types that led to successful fertilisation and early embryogenesis. As the analysis of the embryonic samples took place between days 3 and 6 of development this was prior to any post-implantation selection with regard to embryo viability.

In such carriers of Y autosome translocations, like any typical balanced autosomal reciprocal translocation, a formation of closed ring or open chain type of quadrivalent is expected at meiosis I. Gametes with 2:2 alternate, 2:2 adjacent 1 or 2, or 3:1 or 4:0 modes of segregation may be expected. In the two cases investigated the segregation mode of the male gamete was determined in a total of 27 embryos of which alternate segregation was the most common (53%) followed by adjacent-1 (33%) with a single example of the 3:1 type and no instances of adjacent-2. As is clear from the information in Table 5, there is a significant deficit of certain types of gametes for both the alternate and adjacent-1 modes. The deficit types are the der Y and der autosome combination from the alternate segregation and the X and der autosome combination from adjacent-1. In both translocation cases the deficit groups have in common the der autosome chromosome that includes the segment Yq12 to qter (Fig. 2).

A similar meiotic segregation analysis was performed on spermatozoa from a carrier of 46,X,t(Y;16)(q11.21;q24) translocation. The aim of this analysis was to estimate the risk of using a chromosomally unbalanced spermatozoan during ICSI. Using four-colour FISH, meiotic segregation analysis of 500 sperm revealed that the risk of the combination of chromosomes yielding an unbalanced sperm cell is close to 50%. The most frequent mode of segregation seen in sperm cells was alternate segregation with normal or balanced sperm cells (51%) followed by adjacent-1 (36%) and 3:1 segregation (12%) [10].

Oliver-Bonet and colleagues studied the meiotic behaviour in spermatogenesis of two balanced reciprocal translocation carriers, t(10;14) with normal sperm parameters and t(13;20) with azoospermia. Increased pairing abnormalities, association of the quadrivalent with the sex-body and decreased recombination was seen in the t(13;20) azoospermic carrier whereas in the t(10;14) normozoospermic carrier fewer pairing abnormalities, no association of the quadrivalent with the sex body and a normal frequency of recombination were seen. These observations indicated that pairing abnormalities, association of the quadrivalent with the sex-body and decreased recombination frequency were the possible mechanisms leading to spermatogenic arrest [11]. Similarly, in carriers of Y-autosome translocations involving the heterochromatic Yq12 region, defective spermatogenesis is thought to be most likely due to pairing abnormalities and association of the quadrivalent with sex-body formed during male meiosis [4, 12, 13].

In spermatocytes from Y-autosomal translocation carriers, it was observed that, during the pachytene stage of meiosis I, if a segment of autosome is associated with the sex-body, there is a possibility of it being unpaired or unsynapsed and therefore silenced genetically. It is also thought likely that the sex-body derived inactivation extends to the autosomal segment affecting (silencing) any genes required for the meiotic progression of the spermatocyte, thus leading to degeneration of spermatocytes after the pachytene stage via the pachytene stage checkpoint [12, 13]. Alternatively, it may be that if the autosomal genes required for meiotic progression are not inactivated then the sperm cell will progress through the meiotic prophase but at the time of alignment of the quadrivalent, the presence of asynapsed segments attached to the sex-body will trigger the meiotic spindle checkpoint leading to apoptosis of the sperm cell [14]. Therefore either one of the two mechanisms may be responsible for the arrested spermatogenesis.

Delobel and colleagues studied meiotic configurations at the pachytene stage in testicular biopsies from a carrier with karyotype 46,X,t(Y;6)(q12;p11.1). They clearly observed that in more than three quarters of the cells at pachytene the heterochromatic segment of Y (Yq12 to qter) translocated to the autosome 6 (i.e., der 6) is paired with Xqter, at the PAR2 and is associated with the sex body. In these cells the translocated segment of chromosome 6 is condensed in a similar fashion to the X chromosome. Although they do not comment upon it, critically for the interpretation of our data, close examination of their figures reveals that the translocated segment of the autosome that is attached to the derivative Y is not associated with the sex body and does not appear condensed [12].

To return to the present study, where there is a statistically significant deficit of embryos derived from sperm with the derivative autosome chromosome that includes the segment Yq12 to qter, the most likely explanation may be that this chromosome is associated with the X chromosome at PAR2 in the sex-body leading to inactivation of genes on the autosomal segment that are required for the meiotic process and that this has led to degeneration of this class of spermatocytes during meiosis. Whereas the spermatocytes with the derivative Y chromosome survive because the autosomal segment is not inactivated and genes essential for meiosis are active.

## Conclusion

In carriers of reciprocal translocations the chromosomes involved and position of the breakpoints greatly influence the geometry of the quadrivalent formed at pachytene and hence the segregation types produced. In the particular case of carriers of more rarely occurring Y-autosome translocations other factors such as the association of the heterochromatic region of the chromosome Y (Yqh), bearing the attached segment of the autosome, with the chromosome X via the sex-body during meiosis may affect the expression of genes that are vital for the completion of meiosis (12). This in turn would play an important role in determining the final meiotic outcome and the types of gametes produced.

## Abbreviations

aCGH: Array comparative genomic hybridization
ART: Assisted Reproductive Technology
AZF: Azoospermia factor
FISH: Fluorescence in situ hybridization
ICSI: Intracytoplasmic sperm injection
IVF: In vitro fertilization
PAR: Pseudoautosomal region
PGD: Preimplantation Genetic Diagnosis
SRY: Sex-determining region Y
TE: Trophectoderm
Yqh: Heterochromatic region of the chromosome Y
